# Genetic Networks of Alzheimer’s Disease, Aging, and Longevity in Humans

**DOI:** 10.3390/ijms24065178

**Published:** 2023-03-08

**Authors:** Timothy Balmorez, Amy Sakazaki, Shin Murakami

**Affiliations:** Department of Basic Sciences, College of Osteopathic Medicine, Touro University California, Vallejo, CA 94592, USA

**Keywords:** hallmark of aging, age-related comorbidity, centenarian, dementia, epigenetics, life extension, longevity

## Abstract

Human genomic analysis and genome-wide association studies (GWAS) have identified genes that are risk factors for early and late-onset Alzheimer’s disease (AD genes). Although the genetics of aging and longevity have been extensively studied, previous studies have focused on a specific set of genes that have been shown to contribute to or are a risk factor for AD. Thus, the connections among the genes involved in AD, aging, and longevity are not well understood. Here, we identified the genetic interaction networks (referred to as pathways) of aging and longevity within the context of AD by using a gene set enrichment analysis by Reactome that cross-references more than 100 bioinformatic databases to allow interpretation of the biological functions of gene sets through a wide variety of gene networks. We validated the pathways with a threshold of *p*-value < 1.00 × 10^−5^ using the databases to extract lists of 356 AD genes, 307 aging-related (AR) genes, and 357 longevity genes. There was a broad range of biological pathways involved in AR and longevity genes shared with AD genes. AR genes identified 261 pathways within the threshold of *p* < 1.00 × 10^−5^, of which 26 pathways (10% of AR gene pathways) were further identified by overlapping genes among AD and AR genes. The overlapped pathways included gene expression (*p* = 4.05 × 10^−11^) including ApoE, SOD2, TP53, and TGFB1 (*p* = 2.84 × 10^−10^); protein metabolism and SUMOylation, including E3 ligases and target proteins (*p* = 1.08 × 10^−7^); ERBB4 signal transduction (*p* = 2.69 × 10^−6^); the immune system, including IL-3 and IL-13 (*p* = 3.83 × 10^−6^); programmed cell death (*p* = 4.36 × 10^−6^); and platelet degranulation (*p* = 8.16 × 10^−6^), among others. Longevity genes identified 49 pathways within the threshold, of which 12 pathways (24% of longevity gene pathways) were further identified by overlapping genes among AD and longevity genes. They include the immune system, including IL-3 and IL-13 (*p* = 7.64 × 10^−8^), plasma lipoprotein assembly, remodeling and clearance (*p* < 4.02 × 10^−6^), and the metabolism of fat-soluble vitamins (*p* = 1.96 × 10^−5^). Thus, this study provides shared genetic hallmarks of aging, longevity, and AD backed up by statistical significance. We discuss the significant genes involved in these pathways, including TP53, FOXO, SUMOylation, IL4, IL6, APOE, and CEPT, and suggest that mapping the gene network pathways provide a useful basis for further medical research on AD and healthy aging.

## 1. Introduction

Alzheimer’s disease is the most frequent cause of dementia, in which about 5 million people were living with AD in 2014, and the number is estimated to nearly triple by 2060 [1]. The Center for Disease Control and Prevention (CDC) states that the number of people living with AD doubles every 5 years beyond age 65 (accessed in January 2023; https://www.cdc.gov/aging/aginginfo/alzheimers.htm). Aging and genetic variations are well-known risks for AD. The genetic basis of AD has been characterized in the early onset of AD (EOAD), including amyloid precursor protein (APP) [2], presenilin 1 (PSEN1) [3], and presenilin 2 (PSEN2) [4], which account for less than 1% of AD cases [5]. The vast majority of genetic risk factors fall into late-onset AD (LOAD). The predictive contribution of each gene is little or modest for LOAD [6]. Thus, the gene–gene networks of AD risk factor genes (AD genes) are expected to be more informative than a single gene effect to understand the underlying mechanisms. AD genes identified lipoprotein metabolism as a major hallmark of AD, which is tightly linked to a major mortality risk, cardiovascular disease [7,8]. A component of lipoprotein metabolism includes ApoE isoforms, which are well-known as a risk factor for LOAD [9], as well as associated with longevity [10]. However, although there are genetic variants associated with aging (i.e., pathophysiological changes with increasing age) and longevity (i.e., length of lifespan), genetic interconnections with AD are not yet fully understood [7,8,11]. We reason that studying the gene sets or profiles is an effective way to understand AD and its relationship with aging and longevity. In this study, we chose a type of bioinformatic analysis using Reactome to elucidate the underlying molecular pathways and mechanisms.

Reactome is an open-source bioinformatic database with two functions. Firstly, it is a knowledge database of biological pathways manually curated and peer-reviewed, including twenty-seven biological pathway groups: they are autophagy, cell cycle, cell–cell communication, cellular responses to external stimuli, chromatin organization, circadian clock, developmental biology, digestion and absorption, and disease, among others [12]. The pathway, reaction, and molecule pages are extensively cross-referenced to more than 100 bioinformatics resources, including ChEBI small molecule databases, Ensembl and UniProt databases, NCBI Gene, the UCSC Genome Browser, and PubMed. Secondly, it serves as a bioinformatic tool to perform gene set enrichment analysis (GSEA) [13,14]. GSEA is a computational method to identify and interpret biological functions of a gene set from genome-wide profiles. It uses gene annotations (such as gene ontology (GO), disease ontology (DO), and pathway annotations) and provides a ranked list of annotations with statistical significance for validation of the ranked list [15].

Using the Reactome analysis, we investigated three types of human genes: AD genes, aging-related (AR) genes, and Longevity genes. AD genes have been reported previously, which are validated based on meta-analyses of human GWAS (genome-wide association) studies [7,8,16]. Aging-related (AR) genes in humans are a list of genes identified based on an extensive literature review, followed by manually curated annotation [17]. The genes are from the meta-analysis study of human gene-expression analysis, as well as predicted based on the studies in model systems, including the yeast, the nematode, the fruit fly, and the mouse [17]. The proteomic and genomic map of the AR gene list has been reported earlier [18]. Longevity genes are the genes identified as gene variants associated with longevity [19]. Notable risk factors for Alzheimer’s include APOE, as well as genes associated with inflammation and the insulin/IGF-1 signaling pathway [20]. However, although AD, aging, and longevity may be associated with each other, there is a lack of genetic interaction maps that overview each of them. Thus. We investigated the connections among the genes involved in AD, aging, and longevity.

## 2. Results

**Figure 1:**
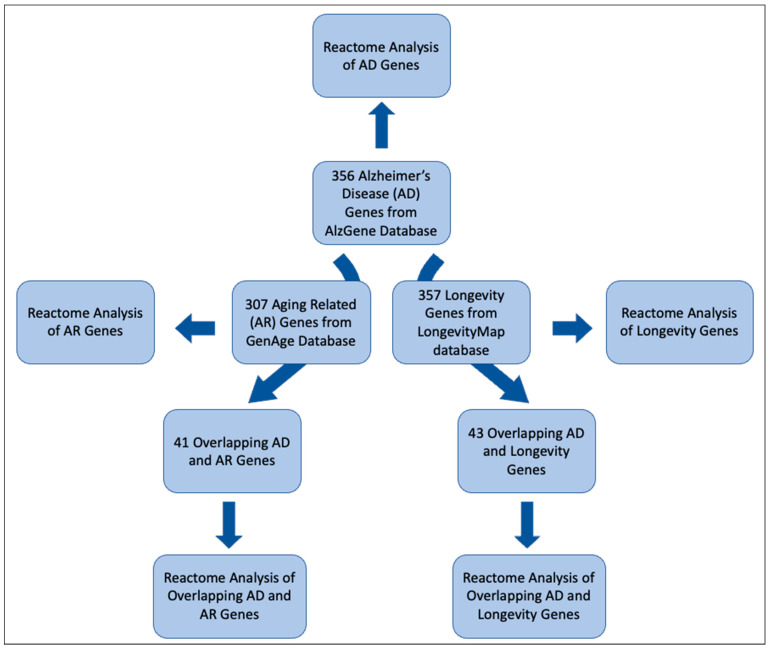
Schematic providing a breakdown of the methodology used in this study. We used the Reactome analysis for AD genes, AR genes, Longevity genes, overlapping AD and AR genes, and overlapping AD and Longevity genes.

Figure 1 summarizes the overall analysis of the gene sets. We identified five gene sets as described in the method. They are (1) 356 positive AD genes, (2) 307 AR genes, (3) 357 Longevity genes, (4) 41 AD–AR overlap genes, and (5) 43 AD–longevity overlap genes (Figure 1). We then perform a Reactome analysis of each gene set that generated biological pathways relevant to the gene set. The pathways were validated by using the threshold of *p* < 10 × 10^−5^; in other words, we excluded the pathways with a false discovery rate of more than 10 × 10^−3^. In each table, we included the top ten results from each Reactome analysis to be included in our results. We further identified specific Reactome groups from within the generalized groups.

### 2.1. AD Genes

The Reactome analysis of the AD genes identified 161 pathways (Supplementary Table S1). Of them, 53 pathways showed *p* < 1.0 × 10^−5^, which is roughly equivalent to the false discovery rate < 1.0 × 10^−3^. The top ten results are shown in Table 1. The pathways can be summarized into the following three general Reactome pathway groups: metabolism of RNA, transport of small molecules, and immune system.

The top hit for the enriched pathway analysis (of AD-positive genes) was “tRNA processing in the mitochondrion” (FDR = 1.01 × 10^−13^; *p* = 1.11 × 10^−16^), which was a sub-pathway topic under “tRNA processing” (FDR = 1.09 × 10^−6^; *p* = 1.09 × 10^−8^), which was a sub-pathway topic under “metabolism of RNA” in the hierarchy panel. “tRNA processing in the mitochondrion” involves 42 proteins, and 19 of those proteins were found to be shared in our positively tested AD genes (FDR = 1.01 × 10^−13^; *p* = 1.11 × 10^−16^). The second top hit was “plasma lipoprotein assembly, remodeling, and clearance” (FDR = 1.34 × 10^−1^; *p* = 4.44 × 10^−16^) from the “transport of small molecules” general pathway. The third-quarters top hit, “Interleukin-4 and Interleukin-13 signaling” (FDR = 5.80 × 10^−10^; *p* = 2.57 × 10^−12^) is a part of the “immune system” general pathway.

### 2.2. AR Genes

669 pathways were identified by the Reactome analysis of the 307 AR genes (Supplementary Table S2). Using the threshold, we validated the pathways and selected 261 pathways using the threshold of *p* < 1.0 × 10^−5^. The top ten results are shown in Table 2. The pathways identified by the AR genes fell into the following six general Reactome pathway groups: immune system, signal transduction, metabolism of proteins, gene expression, cellular responses to external stimuli, and DNA repair. 

The top hits for the enriched pathway analysis and their general pathways were the following: signaling by interleukins (immune system), DNA double-stranded break repair (DNA repair), PIP3 activates AKT signaling (signal transduction), FOXO-mediated transcription (gene expression), cellular responses to stress (cellular responses to external stimuli), SUMO E3 ligases SUMOylate target proteins (metabolism of proteins), cellular senescence (cellular responses to external stimuli), transcriptional regulation by TP53 (gene expression), and SUMOylation (protein metabolism). All results had an FDR = 8.66 × 10^−15^; *p* = 1.11 × 10^−16^. Importantly, AR genes identified SIRT1 and its homologs in five pathways, including “DNA double-stranded break repair”, “FOXO-mediated transcription”, “RNA polymerase II transcription”, “the generic transcription pathway”, and “cellular responses to stress” (Table 2).

### 2.3. Longevity Genes

137 pathways were identified in the Reactome analysis of 357 longevity genes (Supplementary Table S3). Of them, we validated and selected 49 pathways that satisfied the threshold of *p* < 1.0 × 10^−5^. The top ten results are shown in Table 3. The pathways resulted in two general pathways: Signal Transduction and Gene Expression. The top results in the enrichment pathway analysis were part of the “Signal Transduction” general pathway. The Reactome pathway “MTOR signaling”, (FDR = 5.15 × 10^−14^; *p* = 1.11 × 10^−16^) is a sub-pathway topic under “intracellular signaling by second messengers” (FDR = 5.39 × 10^−12^, *p* = 1.74 × 10^−14^). The “MTOR signaling pathway” involves 41 proteins, and 19 of those proteins were found to be shared in our longevity genes. “PIP3 activates AKT signaling” (FDR = 2.39 × 10^−11^, *p* = 1.03 × 10^−13^) is also a sub-pathway topic under the same pathway. In the “gene expression” general pathway, the significant pathways were “RNA polymerase II transcription” (FDR = 1.29 × 10^−9^, *p* = 1.04 × 10^−11^) and two sub-pathways—“TP53 regulates metabolic genes” (FDR = 1.29 × 10^−9^, *p* = 1.11 × 10^−11^) and “FOXO-mediated transcription” (FDR = 1.78 × 10^−9^, *p* = 1.73 × 10^−11^). Longevity genes identified SIRT1 and its homologs in three of the pathways, including “generic transcription pathway”, “RNA polymerase II transcription”, and “FOXO-mediated transcription” (Table 3). The AMPK pathway was identified as “energy-dependent regulation of mTOR by LKB1-AMPK” (Table 3). Both SIRT1/SIRT1 homologs and the AMPK pathway were not found within the categories identified by AD and AD-overlapped genes (i.e., AD–AR and AD–longevity genes).

### 2.4. AD–AR Overlap Genes

41 AR genes (13% of the genes) overlapped with AD genes (AD–AR overlap genes) (Table 4). The Reactome analysis of the AD–AR overlap genes showed 261 pathways (Supplementary Table S4). Of them, we validated and selected 24 pathways that satisfied the threshold of *p* < 1.0 × 10^−5^. The top ten results are shown in Table 5. The pathways resulted in five general Reactome groups: gene expression, metabolism of proteins, programmed cell death, signal transduction, and immune system.

The top result for the enriched pathway analysis was “Generic Transcription Pathway” (FDR = 1.85 × 10^−8^; *p* = 4.05 × 10^−11^), which was a sub-pathway topic under “RNA polymerase II transcription” (FDR 1.85 × 10^−8^; *p* = 2.84 × 10^−10^), which was a sub-pathway topic under the “Gene Expression” general pathway. Within “Generic Transcription Pathway”, the analysis highlighted “nuclear receptor transcription pathway” (FDR = 9.705 × 10^−4^; *p* = 4.85 × 10^−5^) and “TP53 regulates transcription of cell death genes” (FDR = 5.90 × 10^−4^; *p* = 2.36 × 10^−5^). “Generic transcription pathway” involves 1234 proteins and 22 of those proteins were found to be shared in AD–AR overlap genes (FDR = 1.85 × 10^−8^; *p* = 4.05 × 10^−11^).

**Figure 2:**
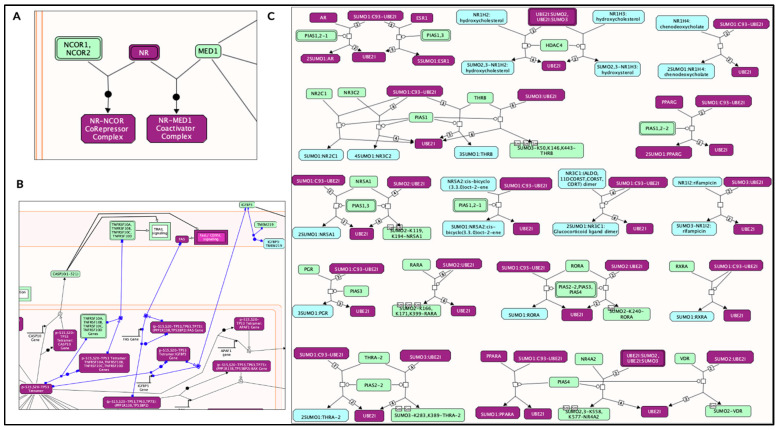
Enriched pathway analysis. The entities colored purple are hits within the positive gene list. Proteins are rectangles whereas elongated hexagons are complexes. (**A**) Generic transcription pathway. (**B**) TP53 Regulates the transcription of death receptors and ligands. (**C**) SUMOylation of intracellular receptors.

**Figure 3:**
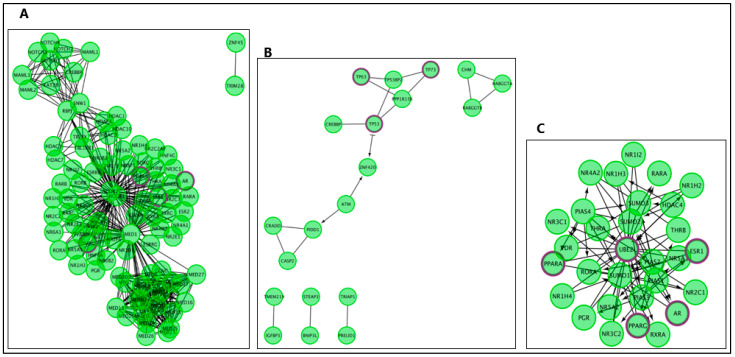
The Reactome pathways from Figure 2 converted into a functional interaction network. Sub-pathways within the original pathway diagrams were extracted into the FI network as well. Hit genes are displayed in a thick purple border in the FI network view for a hit pathway. (**A**) Generic transcription pathway. (**B**) TP53 regulates the transcription of death receptors and ligands. (**C**) SUMOylation of intracellular receptors.

Pathways within the AD–AR overlap genes (Figure 2 and Figure 3) for the “generic transcription pathway” included the following sub-pathways: “nuclear receptor transcription pathway” and “transcriptional regulation by TP53”. Under “SUMOylation” hit genes included the following sub-pathways: “SUMO E3 ligases SUMOylate target proteins”, “SUMOylation of intracellular receptors”, “SUMOylation of transcription factors”, and “SUMOylation of DNA damage response and repair proteins”.

### 2.5. AD–Longevity Overlap Genes

43 genes of longevity genes (12% of the genes) overlapped with AD genes (Table 6). The Reactome analysis of the AD–longevity overlap genes identified 34 pathways (Supplementary Table S5), of which 12 pathways were validated within the threshold of *p* < 1.0 × 10^−5^. The top ten results are shown in Table 7. The pathways were in the following 4 Reactome general pathways: Immune System, Plasma lipoprotein assembly, remodeling, and clearance, Metabolism of vitamins and co-factors, and Signal Transduction.

**Figure 4:**
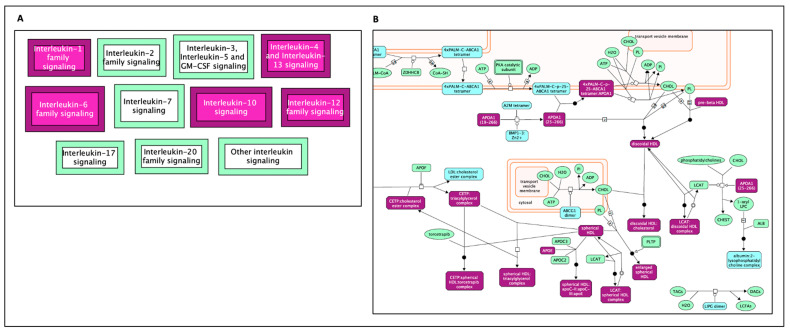
Enriched pathway analysis. The entities colored purple are hits within the positive gene list. Proteins are rectangles whereas elongated hexagons are complexes. (**A**) Generic transcription pathway. (**B**) TP53 regulates the transcription of death receptors and ligands.

**Figure 5:**
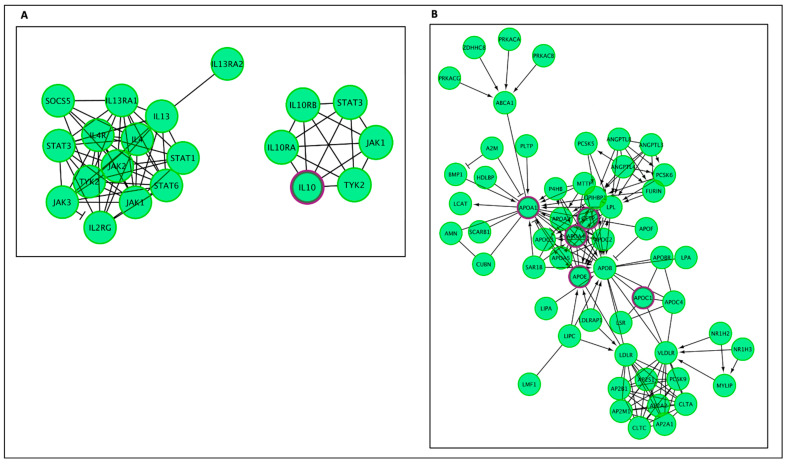
The Reactome pathways from Figure 4 converted into a functional interaction network. Hit genes are displayed with a purple border. (**A**) FI network for the diagram of Interleukin-4, Interleukin-13, and Interleukin-10 signaling, (**B**) FI network for the diagram of plasma lipoprotein assembly, remodeling, and clearance.

The top result from the enriched pathway analysis is “Interleukin-4 and Interleukin-13 signaling” (FDR = 2.32 × 10^−5^; *p* = 7.64 × 10^−8^), which is a sub-pathway of “signaling by Interleukins” (FDR 4.57 × 10^−4^; *p* = 1.20 × 10^−5^) and part of the “Immune System” general pathway. Out of the 112 proteins found to be involved in the “Interleukin-4 and Interleukin-13 signaling” pathways, 7 were shared by AD–longevity overlap genes. Key hit genes involved with these pathways include TNF, IL10, IL18, and IL6. These genes are also shown to be involved in Interleukin 10 signaling. Interleukin pathways are shown in Figure 4A and Figure 5A.

The second major Reactome group is “Plasma lipoprotein assembly, remodeling, and clearance” (FDR = 2.30 × 10^−4^; *p* = 3.69 × 10^−6^). Genes in this pathway (Figure 4B and Figure 5B) incorporate the following significant sub-pathways: “plasma lipoprotein assembly” (FDR = 7.82 × 10^−5^, *p* = 5.15 × 10^−7^), which is a sub-pathway of “plasma lipoprotein remodeling“, “chylomicron remodeling”, “chylomicron assembly”, and “HDL remodeling”. The hit genes CEPT and APOE are present in all of these subcategories, highlighting their importance in this AD and longevity overlap dataset. Other top results included “Retinoid metabolism and transport”, which is a subset of “Metabolism of fat-soluble vitamins” and “NR1H2 and NR1H3-mediated signaling”, a subset of the “signal transduction” pathway. APOE is also a hit gene in these pathways.

## 3. Discussion

**Figure 6:**
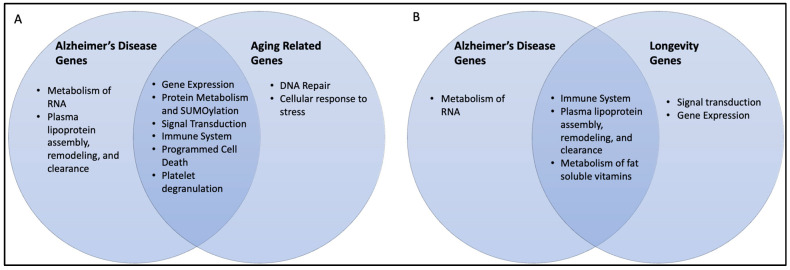
Venn diagrams of overlapping pathways: (**A**) Alzheimer’s genes and aging-related genes and (**B**) overlapping Alzheimer’s genes and longevity genes. The pathways shown in Table 1, Table 2, Table 3, Table 5 and Table 6 were used.

This study identified a diverse range of biochemical pathways, using the Reactome analysis of each individual set of AD, AR, and longevity genes. We compiled and removed redundancies of the pathways into comparable groups by highlighting the hallmarks of each subset and comparing each individual gene set to the pathways involved in the overlapping (AD–AR and AD–longevity) gene sets. Figure 6 summarizes pathways involved in each subset, which are further discussed in the subsequent sections.

### 3.1. AD Genes

**Figure 7:**
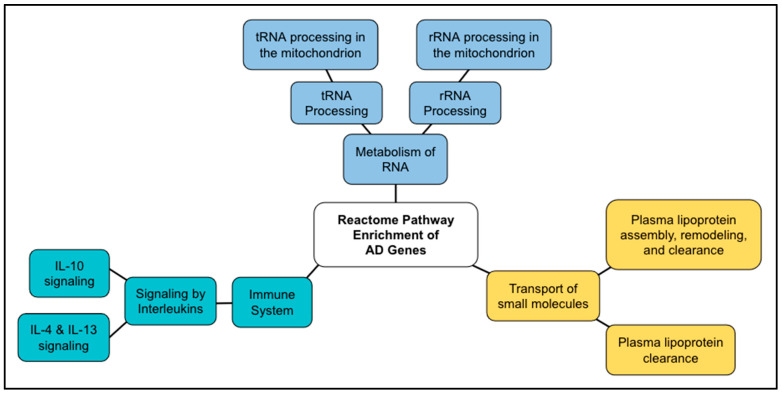
Diagram of the Reactome pathway enrichment of AD genes. The results shown in Table 1 are summarized.

The AD genes are associated with the health risk of AD, which were identified previously [7,8]. The top pathways (Table 1) were grouped into three categories: mitochondrial RNA metabolism (processing of RNA), lipoprotein metabolism (lipid metabolism), and interleukin signaling (immune system) (Figure 7). The pathway of “Mitochondrial RNA metabolism” was unique to the pathways identified by AD genes, while the other two categories, lipoprotein metabolism (lipid metabolism) and interleukin signaling (immune system), also overlapped with the pathways identified by Longevity genes. We discuss mitochondrial RNA here, and the others are discussed below in Section 3.4.

Mitochondrial RNA metabolism aligns with the mitochondrial cascade that controls mitochondrial function and change rates influence AD chronology. Reactome analysis has made mitochondrial genes the top hit when associated with AD genes alone, signaling its importance and the need for more data on possible interventions. Mitochondrial RNA metabolism falls into diverse functions of mitochondrial complexes, tRNA, and rRNA. Although the roles of mitochondrial RNA in AD remain unclear, mitochondrial dysfunctions and cascades have been involved in AD pathogenesis, thus providing potential markers for neurodegeneration [21,22,23,24]. The mitochondrial coding and non-coding RNA are increased in the circulating extracellular vesicles in AD and mild cognitive impairment (MCI) [25,26]; the AD genes in the category of this study include MT-ND1-4,6, MT-ND4L, MT-ATP6, MT-ATP8, MT-CYB (or MT-CYBT), MT-CO1, and MT-RNR1 (Table 1). Alterations in the mitochondrial RNA generally impact energy-demanding tissues, including the brain and the muscle [27,28], and the mitochondrial instability in the neurons, releasing mitochondrial RNAs in the circulating vesicles in the blood [25,26]. Deficiencies of the RNA genes are also related to AD; Parkinson’s disease; and a variety of neurological and mitochondrial diseases, including mitochondrial myopathy, encephalopathy, lactic acidosis, stroke-like episodes (MELAS), Leber hereditary optic neuropathy, myoclonic epilepsy associated with ragged-red fibers (MERRF), and Leigh syndrome [29,30,31,32].

### 3.2. AR Genes

**Figure 8:**
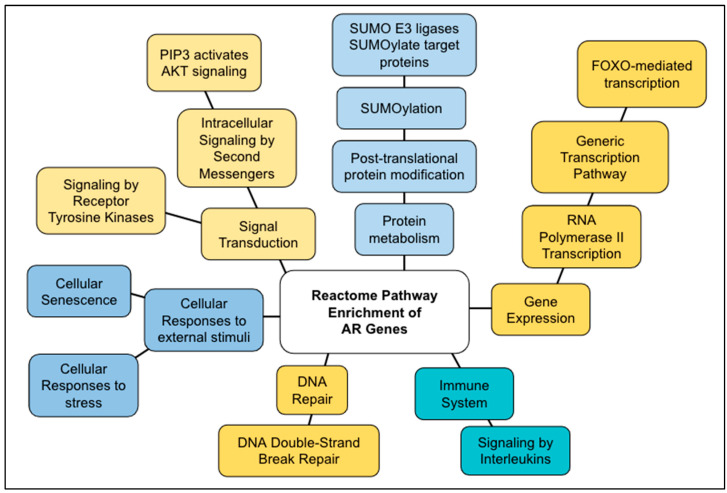
Diagram of the Reactome pathway enrichment of AR genes. The results shown in Table 2 are summarized.

The AR genes are associated with aging. The top pathways (Table 2) can be grouped into six general pathways (Figure 8). Of them, the genes unique to the AR subset are components of two pathways, DNA double-stranded break (DSB) repair (30 genes) and cellular senescence (61 genes) (Table 2, Figure 6). DSB can precipitate genomic rearrangements affecting multiple genes thus leading to much broader consequences when compared to other types of DNA mutations. This suggests that as humans age, DSB, and other DNA repair mechanisms become less efficient and more error-prone [33]. Deficiencies in the DSB pathway are known to be involved in breast and colorectal cancer [34]; xeroderma pigmentosum [35,36]; Werner syndrome [37]; and various DSB syndromes, including Fanconi anemia, Nijmegen breakage syndrome, and ataxia–telangiectasia [38,39], among others.

Cellular senescence, or cellular aging, is a cellular response characterized by stable growth arrest, altered proinflammatory secretome, and other phenotypic alterations. The alterations are known to play a role in normal development, tissue homeostasis, and preventing tumor progression [40]. It has been implicated as a major cause of age-related disease. This suggests that the further study of cellular senescence can lead to novel therapies for age-related diseases. The genes in cellular senescence are involved in a wide variety of cancers (hepatocellular carcinoma and colorectal, breast, prostate, ovarian, and lung cancers) [41,42]. Notably, cellular senescence may be involved in the defense against cancer.

### 3.3. Longevity Genes

**Figure 9:**
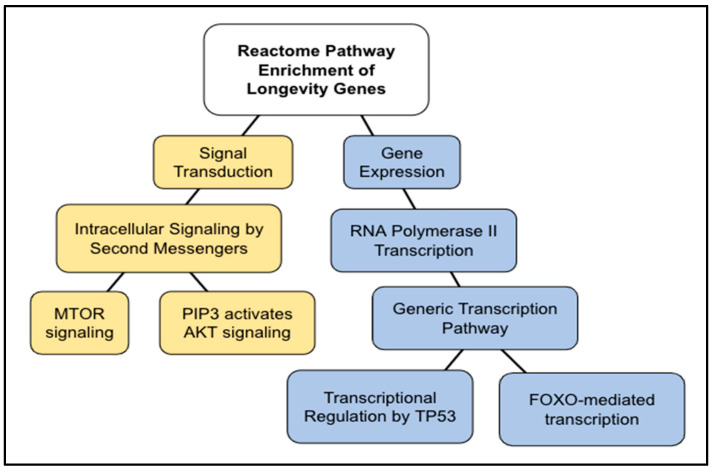
Diagram of the Reactome pathway enrichment of longevity genes. The results shown in Table 3 are summarized. Two main pathways are shown out of twelve pathways identified.

The longevity genes are associated with increased lifespan, which is an indicator of delayed aging. The top pathways (Table 3) can be grouped into two pathways: the signal transduction category (PIP3-AKT and mTOR pathways) and the gene expression category (TP53 and FOXO pathways) (Figure 9). The TP53 and FOXO pathways are discussed elsewhere (Section 3.5). PIP3-AKT is a part of the insulin/IGF-1 signaling (IIS) pathway, which regulates FOXO (forkhead box O), a subset of a large family of transcription factors. The insulin/IGF-1 pathway is known to control lifespans and stress resistance to protect against multiple forms of stress damage in model systems [43,44,45,46,47,48,49]; the other life-extending pathways include the Sirtuin/SIRT1 pathway, the mTOR pathway, and the AMPK pathway, among others in model systems [50]. Consistently, SIRT1 and its homologs were involved in five of the top AR pathways (Table 2) and three of the top Longevity pathways (Table 3), while the AMPK pathway was also involved in Longevity genes (Table 3).

The IIS pathway is also involved in cellular processes such as cell growth and survival, including FOXO3 and FOXO1 genes [51]. The mechanistic target of rapamycin (mTOR), which is also a downstream target of the insulin/PIP3/AKT pathway, is a protein kinase in a highly conserved pathway that senses nutrients and other environmental signals and coordinates several fundamental cellular responses, such as cell growth and proliferation, and has been linked to the physiological process of aging. [52]. Previous studies have shown that inhibition of mTOR enhances longevity and decreases aging and age-related disease in model organisms [53]. The transcription factor TP53, which encodes tumor suppressor p53, is involved in several aging-related pathways such as apoptosis and senescence and has also been shown to influence insulin/mTOR signaling, which can contribute to longevity [54].

### 3.4. Comparing AR Pathways with Longevity Pathways

**Figure 10:**
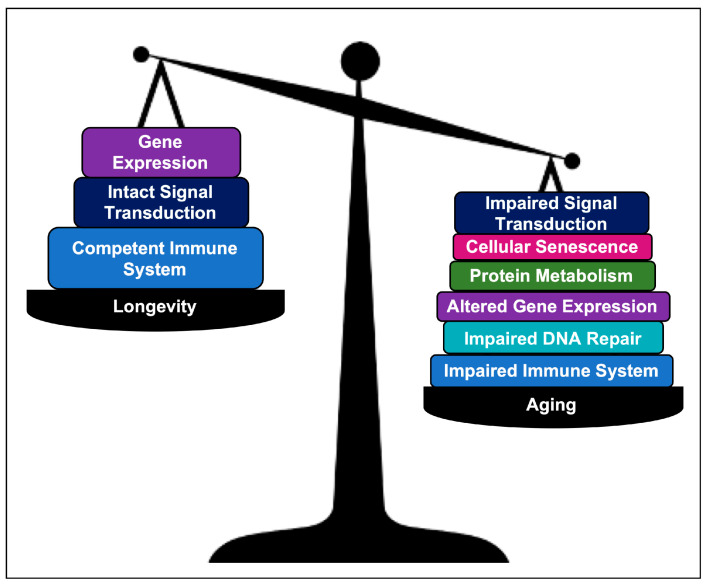
A schematic illustrating proposed genetic pathways that contribute to aging and longevity.

Based on our Reactome analysis, there are shared biological pathways among AR and longevity genes, including the immune system and cytokine signaling, signal transduction, and gene expression, involved in the balance between aging and longevity. Aging also has components of impaired DNA repair and metabolism of proteins, as well as cellular senescence. We have compared the pathways involved in AR and longevity genes, which represent the balance of shared pathways (Figure 10).

### 3.5. AD–AR Overlap Genes

**Figure 11:**
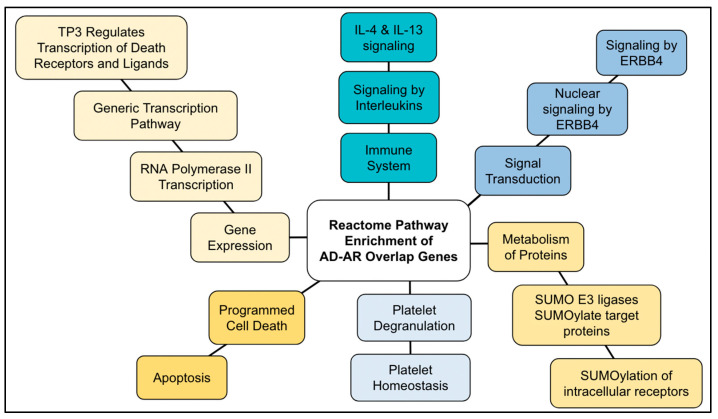
Diagram of the Reactome pathway enrichment of AD–AR overlap genes. The results shown in Table 5 are summarized.

Although the gene ontology for the AD–AR overlap genes demonstrated involvement in a broad range of biological pathways, concurrent with previous studies, some of our most significant associations involve TP53, FOXO, and SUMOylation (Table 4 and Figure 11). The AD and AR genes have a positive trajectory to aging, which may be involved in creating the ground of both aging and AD.

The TP53 gene, or the gene product p53, is well known for its guardian function as a prominent tumor suppressor protein that protects genome integrity through cell cycle control and the DNA damage response, among others. In neurons, p53 has pleiotropic functions. AD brain pathology shows abnormal cell cycle and apoptosis and increased DNA damage, all of which are dependent on p53 [55]. In addition, misfolded p53 has been observed in MCI and AD [56]. p53 interacts with beta-amyloid and tau [57,58] and forms aggregates to form oligomers and fibrils and interacts with tau oligomers [55]. The p53 protein is degraded and inactivated to a ubiquitin-dependent pathway [59]. In AD, protein degradation pathways, the ubiquitin–proteosome system, and autophagy are reduced, leading to defective proteostasis [60]. Thus, trajectories of the p53 functions are both positive and negative for AD and aging. It has been proposed that p53 is a potential peripheral biomarker that could detect AD at its earliest stages [56].

FOXO proteins are a subgroup of the Forkhead family of transcription factors involved in the regulation of aging and stress resistance, metabolism, regulation of reactive species, and regulation of cell cycle arrest and apoptosis. The FOXO family includes DAF-16 in *Caenorhabditis elegans* and dFOXO in *Drosophila melanogaster*, as well as mammalian FoxO1, FoxO3, FoxO4, and FoxO4. FOXO has diverse functions, including Lipid metabolism, DNA damage repair, stress resistance, autophagy, and glucose metabolism, among others [43,47,61,62]. Consistently, in mice with FOXO3 deficiency, there is an increased plaque load and core plaque size, commonly found in AD progression, in the cortex [63]. Importantly, p53 and FOXO are both transcription factors, which can function through interlocking pathways involving SIRT1 and microRNA [64].

### 3.6. AD–longevity Overlap Genes

**Figure 12:**
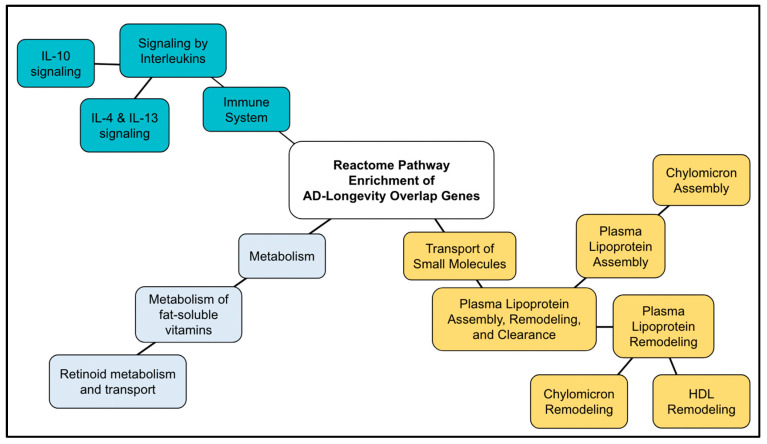
Diagram of the Reactome pathway enrichment of AD–longevity overlap genes. The results shown in Table 6 are summarized.

Significant pathways generated from the AD–longevity overlap set include three pathways, cytokine signaling in the immune system, lipoprotein metabolism, and the metabolism of fat-soluble vitamins and NR1H2- and NR1H3-mediated signaling (Figure 12). The latter two pathways are closely related to each other, since lipoproteins are a carrier of fat-soluble vitamins and metabolism, and thus, we combined them as a lipoprotein metabolism. As discussed above, AD genes identified mitochondrial RNA metabolism, lipoprotein metabolism, and cytokine signaling. Mitochondrial RNA metabolism was identified by AD genes but not longevity genes, suggesting that it is unique to AD genes, while lipoprotein metabolism and cytokine signaling were also identified by longevity genes, which are likely involved in a more general basis in longevity (i.e., delayed aging). Although it remains unclear, the AD–longevity overlap genes are candidates that may be involved in the gene–gene network protective to healthy aging.

In the first category immune system, cytokines play an integral role in regulating inflammatory pathways, including neuroinflammation in Alzheimer’s disease. Aß plaques have been shown to increase levels of proinflammatory cytokines IL6 and TNF alpha, among others, leading to a vicious cycle of cytokine-derived inflammation and plaque accumulation [65]. Proinflammatory cytokines, including TNF alpha and IL6, generally become more prevalent with age [66,67,68]. Genetic differences in the cytokine genes could serve as a potential connection to either longevity or aging and inflammatory age-related diseases [69]. It has been theorized that in longer-lived individuals, the polymorphisms that express lower IL6 levels would be negatively correlated, but studies have had mixed results [70,71,72]. IL4 is an anti-inflammatory in the brain and has been shown to potentially counter the inflammatory processes in age-related diseases in mice, although detailed mechanisms are not yet known [73]. Our present study reveals the importance of the interplay and balance of cytokines, especially IL4 and IL6, which would be a potential area for further research. Importantly, low-grade increase in age-related inflammation has led to two hypotheses: the molecular changes cause age-related inflammation (molecular inflammation hypothesis) [68], and age-related progressive increase in proinflammatory status cause age-related inflammation (inflammaging hypothesis) [67,74]. However, the two hypotheses are not sufficient to explain age-related chronic inflammation [68] caused by, for example, a stress response and senescent-associated inflammatory response through secretome cytokines (discussed below).

The lipoprotein metabolism was the second category in the AD–longevity overlap genes. A lipoprotein metabolism associated with AD has been discussed previously [7,8]. Briefly, the human brain is a lipid-rich tissue that contains 35–80% of lipids [75,76]. Lipoproteins transport lipids and lipid-soluble compounds (e.g., lipid-soluble vitamins A, D, E, and K), controlling lipid homeostasis in the body. Thus, it is reasonable to assume the role of lipoprotein in the brain. Essential fatty acids (EFAs) are required for brain development and maintenance. EFAs are also precursors of inflammatory mediators, eicosanoids, which are known to be involved in the pathology of beta-amyloid [77,78]. In this study, the Reactome analysis identified 21 genes (Table 1) in the pathway of lipoprotein metabolism. In this study, the Reactome analysis identified 21 genes, including Apolipoprotein E (APOE) and cholesteryl ester transfer protein (CEPT) (Table 1), that fell into the Reactome pathway of plasma lipoprotein assembly, remodeling, and clearance. Polymorphisms of APOE are an established risk factor for developing AD by enhancing Aß-led inflammation and deterioration [79]. Polymorphisms of CEPT are potential candidates for risk factors for developing AD, but results have been discordant [80]. Despite being shown to increase the risk of developing AD, these genes are difficult to target or change, since they are needed for normal lipid homeostasis. Further, variants could be evolutionarily beneficial earlier in life, an example of antagonistic pleiotropy [81]. Thus, future studies should emphasize the interplay between genes and lifestyle throughout a person’s lifetime.

Interestingly, stress damage and response interlock the immune system and lipoprotein metabolism in multiple ways. Some examples are (1) oxidative damage to low-density lipoproteins (LDL) is a well-known initial step of atherosclerosis, inducing inflammatory responses by releasing cytokines and chemokines [82,83,84]. (2) Metabolically linked proinflammatory stress, such as endoplasmic reticulum (ER) stress, modulates lipid metabolism and mediates the release of cytokines (IL-1α, IL-6, and IL-8), which are known to participate in age-related chronic inflammation [68]. ER stress triggers an unfolded protein response (UPR) and transcriptionally regulates lipogenesis [85], thus playing an essential role in lipid metabolism [85,86,87,88]. (3) Stress damage such as DNA damage and response play a major role in cellular senescence in vitro [89]. Cellular senescence triggers a potent senescent-associated inflammatory response through secretome cytokines, IL-1β, IL-6, and IL-8 [90,91]. Recent studies suggest a relationship between the lipid metabolism and senescence [92,93]. Oxidative stress and cellular senescence are involved in age-related comorbidities, including cardiovascular disease, chronic kidney disease, diabetes, neurodegenerative diseases, and macular degeneration, among others [94]. Cardiovascular (CV) risk factors (i.e., obesity, diabetes, hypertension, and atherosclerosis) are associated with the inflammatory pathway mediated by IL-1α, IL-6, and IL-8 [95].

### 3.7. Technical Advantages and Limitations

Gene set enrichment analysis (GSEA) is a computational method that is useful to interpret the biological functions of a gene set with statistical confidence. Reactome incorporates GSEA embedded with the knowledge database that covers more than 100 bioinformatics resources. The Reactome outputs include statistical confidence levels with *p*-values and false discovery rate, which raises the confidence level of evidence. The gene enrichment algorithms use a score-ranked list and compare it with random rankings (Subramanian et al., 2005), which provides a reasonable level of statistics with a *p*-value and false discovery rate. Thus, we suggest that the results from this study represent the most current genetic hallmarks involved in AD, aging, and longevity. This study provides a comprehensive update of the genomic map of the AR genes reported nearly two decades ago [18].

However, the results from GSEA should be viewed as living knowledge, which requires updates until the knowledge is saturated and complete. A potential limitation is that connections among genes and annotations may be influenced by outliers among previous studies, which should be minimized by peer review, systematic review, and meta-analysis. Although poor study design and controls in a GWAS study may influence overall annotations, it is likely that GSEA and the review systems reduce the impact of such a study. We also used a stringent condition (*p* < 1.00 × 10^−5^), which is backed up by the false discovery rate < 1.00 × 10^−3^. Additionally, knowledge databases, such as Reactome (https://reactome.org/) and KEGG (https://www.genome.jp/kegg/), process the data rigorously with continued updates, which should reduce the incorporating experimental errors. Another limitation is manually curated annotation nomenclatures, some of which may not be easy to understand. For example, stress resistance to a variety of stresses (i.e., multiplex stress resistance) is a component of longevity [43,44,96], which cannot be identified using the current annotation and thus remains to be investigated.

Another limitation comes from the gene set enrichment analysis (GSEA) that identifies gene hits within a group of genes in a particular pathway supported by statistical confidence (*p*-value and FDR). GSGA is a powerful method to identify and overview pathways that come with statistical confidence and minimum bias since a single-gene effect is not necessarily predictable to define phenotypes and trajectories. For example, the TP53 gene also shows both positive and negative trajectories: p53 as guardian of the genome is positive for longevity, while misfolded p53 observed in the background of MCI and AD has negative trajectories against longevity (discussed in Section 3.5). There are traits, such as IP6K3 and IPMK, that show controversial phenotypes compared to the overall gene–gene effects, suggesting that gene–gene interactions may be more important than single polymorphisms [97]. Lastly, the insulin/IGF-1 pathway, including PIP3-AKT, is positive for longevity when the function is reduced, while it is lethal when the function is knocked out (Section 3.3). For these reasons, it is important to assess interactions among genes and variants to define different phenotypes. This study using GSGA provides a general overview of major pathways as a blueprint of hallmarks relevant to phenotypic and disease ontology (e.g., AD, aging, and longevity in this study), while a personalized genetic overview, or direct-to-consumer genetic application for the assessment of health risks, would require a multidimensional analysis with genetic contributions of variants, epistatic effects, phenotypic and disease ontology, and other nongenetic effects in an individual [8,97,98,99,100,101].

## 4. Materials and Methods

### 4.1. Datasets

We used three sets of genes for our study: human Alzheimer’s disease (AD) genes, aging-related (AR) genes, and longevity genes. With these three gene sets, two additional gene sets were created by: (1) identifying the genes shared both by the AD genes and by the AR genes (AD–AR Overlap) and (2) identifying the genes shared both by AD genes and longevity genes (AD–longevity overlap). Firstly, we utilized the AlzGene database (www.alzgene.org; last accessed 10 May 2021) to extract a list of 680 identified human AD genes from GWAS and previous linkage studies [16,102]. Each gene from the database was linked to a number of positive or negative test results from each GWAS study, which were then used to validate the reliability of the data. Out of the 680 genes, 356 genes had positive results (AD genes), and 324 genes had negative results. Since the definite negative and positive results were not included in the database, we used the *p*-value from previous studies for each gene. If *p* < 0.05, we assumed the data was reliable and counted them as a positive result; studies with *p* > 0.05 were regarded as negative and were not included in our study. Note that we used a more stringent threshold of *p* < 1.0 × 10^−5^ to validate the Reactome pathways (see text). Research outcomes listed as trends or inconclusive were also not included in our study. Secondly, we utilized the GenAge database (www.genomics.senescence.info; last accessed 10 May 2021), a benchmark database of 307 genes possibly related to human aging (AR). The genes were extensively reviewed for inclusion based on findings in the model organisms put in the context of human biology, plus genes directly related to aging in humans [103]. Thirdly, we utilized the LongevityMap database (www.genomics.senescence.info/longevity/; last accessed 12 December 2021), a database of 751 genes associated with human longevity [20]. In the database, 394 genes were labeled “non-significant” and thus excluded from this study for analysis. The remaining 357 genes were labeled as “significant” (Longevity genes) and used in this study. Finally, the list of AD Genes was used to cross-reference the presence of any genes shared by the combinations of each gene set (AD–AR overlap genes and AD–longevity overlap genes). When comparing AD genes with AR genes, 41 overlapping (AD–AR overlap) genes were found. When comparing AD genes and Longevity genes, 43 overlapping (AD–longevity overlap) genes were found.

### 4.2. Gene Ontology: Reactome Analysis

We used Reactome (www.reactome.org) to analyze the pathways involved in each gene set. The pathways with a threshold of *p* < 10 × 10^−5^ were selected and used for this study. Reactome analysis was performed as described previously [7,8]. Reactome FIViz was used to determine enrichment in the Functional Interaction (FI) network, the pathway enrichment of the genes of interest, followed by converting the results to interactomes. Statistics and false discovery rate (FDR) were calculated by the Reactome FIViz. We used Cytoscape ver. 3.8.2 (Java version: 11.0.6) to run the Reactome software plugin, Reactome FIViz app [104]. The version of the pathway database was Reactome v76 (released on 21 March 2021; last accessed on 26 May 2021).

## 5. Conclusions and Future Direction

Although a large number of genetic studies on aging have made major findings in aging and longevity, how the findings may apply to AD was unclear. In this study, using human gene sets, we have successfully identified and overviewed the gene–gene networks of aging and longevity and their association with Alzheimer’s disease genes. The Reactome analysis used in this study provides the genetic pathways with gene set enrichment and statistical confidence levels. Our results suggest overlapping pathways that involve TP53, FOXO, protein metabolism (SUMO), mitochondrial RNA metabolism, cytokine balance, and lipoprotein metabolism, among others. The genetic hallmarks identified in this study provide unexpectedly broad mechanisms, suggesting a wide variety of implications in the field of aging. Importantly, AD genes are associated with a variety of pathways that link to age-related comorbidities, thus providing a view in which AD genes create the ground of age-related comorbidities [8], particularly mitochondrial RNA metabolism, cytokine signaling, and lipid metabolism with the highest significance. Gene profiling-based treatment would distinguish age-related comorbidities specific to AD and nonspecific to AD. Further studies on environmental and lifestyle factors may provide a genetic and epigenetic understanding of the development of Alzheimer’s disease and age-related comorbidities [101,105]. Moreover, AD genes are also associated with common age-related comorbidities, including diabetes, myocardial infarction, heart disease, hypertension, cardiovascular system disease, and vascular disease [8]. Importantly, there is an inverse relation between AD and cancers [8,106]. More details have been discussed previously [7,8]. The genetic network in this study should contribute as a blueprint for a personalized genetic risk assessment for AD and other age-related comorbidities (Section 3.7). We also note the technical limitation that manually curated annotations in Reactome may need to be optimized to understand AD, aging, and longevity more accurately. Research such as this study on the genetic network is expected to link aging, mid-life common diseases, and Alzheimer’s disease [107,108]. We suggest that the overviews of the gene sets, including those in this study, continue to be essential for the understanding of AD, aging, and longevity for the following reasons: (1) The predictive contribution of each genetic variant remains modest for LOAD [6]. Previous findings on single-gene effects of human genes have limitations that may miss the complexity of human genetics. Thus, gene–gene networks should be considered. (2) There are genes with controversial phenotypic effects [97]. The prediction has been supported by the observation that the genetic network and epistasis analysis estimates genetic effects better than single-gene effects [8,97,101].

Previously, we have proposed to include feedback from patients in research when studying health [109] and provided an example [110]. We incorporated their feedback that there might be a more meaningful approach to AD patients than simply identifying the AD genes. The discussion prompted us to explore related genes and extracted the biological hallmarks shared among aging, longevity, and AD through outreach and education within the local, national, and scientific communities [109]. The genetic interaction networks among aging, longevity, and AD provide the extraction and translation of the gene information into the hallmarks as well, as are the key to developing effective treatments for AD. We hope to stimulate basic science research open to patients, the community, and education.

## Figures and Tables

**Table 1:** Reactome pathway enrichment analysis results—Positive AlzGene (AD) genes.

Reactome Pathway	No. of Total Proteins in Pathway	No. of Hits in Pathway	*p*-Value	FDR Value	Hit Gene
tRNA processing in the mitochondrion	42	19	1.11 × 10^−16^	1.01 × 10^−13^	*MT-ND6*, *MT-TQ*, *MT-ND4L*, *MT-ND4*, *MT-CO1*, *MT-TT*, *MT-TR*, *MT-ND2*, *MT-ND3*, *MT-ND1*, *MT-TH*, *MT-CO2*, *MT-TG*, *MT-CO3*, *MT-TS2*, *MT-ATP6*, *MT-ATP8*, *MT-RNR1*, *MT-CYB*
Plasma lipoprotein assembly, remodeling, and clearance	71	21	4.44 × 10^−16^	1.34 × 10^−13^	*LIPA*, *LIPC*, *SOAT1*, *CETP*, *APOE*, *A2M*, *ABCA1*, *VLDLR*, *LDLR*, *NR1H2*, *ABCG1*, *LPL*, *ALB*, *APOA1*, *APOA4*, *APOA5*, *NPC1*, *NPC2*, *APOC4*, *APOC2*, *APOC1*
rRNA processing in the mitochondrion	38	17	4.44 × 10^−16^	1.34 × 10^−13^	*MT-ND4L*, *MT-ND4*, *MT-CO1*, *MT-TT*, *MT-TR*, *MT-ND2*, *MT-ND3*, *MT-ND1*, *MT-TH*, *MT-CO2*, *MT-TG*, *MT-CO3*, *MT-TS2*, *MT-ATP6*, *MT-ATP8*, *MT-RNR1*, *MT-CYB*
Interleukin-4 and Interleukin-13 signaling	112	21	2.57 × 10^−12^	5.80 × 10^−10^	*ICAM1*, *TP53*, *MAOA*, *PIK3R1*, *HMOX1*, *CD36*, *IL10*, *IL18*, *IL1A*, *IL1B*, *PTGS2*, *ALOX5*, *F13A1*, *TNF*, *TGFB1*, *POU2F1*, *IL6*, *IL8*, *MMP1*, *MMP3*, *CCL2*
Plasma lipoprotein clearance	33	12	9.58 × 10^−11^	1.73 × 10^−8^	*LIPA*, *LIPC*, *SOAT1*, *APOE*, *VLDLR*, *LDLR*, *NR1H2*, *APOA1*, *NPC1*, *NPC2*, *APOC4*, *APOC1*
Interleukin-10 signaling	47	13	4.28 × 10^−10^	6.46 × 10^−8^	*IL1RN*, *ICAM1*, *CCR2*, *IL10*, *IL18*, *IL1A*, *IL1B*, *PTGS2*, *TNF*, *IL6*, *IL8*, *CCL3*, *CCL2*
Retinoid metabolism and transport	44	12	2.36 × 10^−9^	3.05 × 10^−7^	*LRAT*, *HSPG2*, *APOE*, *LDLR*, *LPL*, *APOA1*, *APOA4*, *LRP1*, *LRP2*, *LRP8*, *TTR*, *APOC2*
Metabolism of fat-soluble vitamins	48	12	6.14 × 10^−9^	6.93 × 10^−7^	*LRAT*, *HSPG2*, *APOE*, *LDLR*, *LPL*, *APOA1*, *APOA4*, *LRP1*, *LRP2*, *LRP8*, *TTR*, *APOC2*
tRNA processing	146	19	1.09 × 10^−8^	1.09 × 10^−6^	*MT-ND6*, *MT-TQ*, *MT-ND4L*, *MT-ND4*, *MT-CO1*, *MT-TT*, *MT-TR*, *MT-ND2*, *MT-ND3*, *MT-ND1*, *MT-TH*, *MT-CO2*, *MT-TG*, *MT-CO3*, *MT-TS2*, *MT-ATP6*, *MT-ATP8*, *MT-RNR1*, *MT-CYB*
Plasma lipoprotein remodeling	32	10	1.47 × 10^−8^	1.32 × 10^−6^	*LIPC*, *CETP*, *APOE*, *ABCG1*, *LPL*, *ALB*, *APOA1*, *APOA4*, *APOA5*, *APOC2*

**Table 2:** Reactome pathway enrichment analysis results—GenAge (AR) genes.

Reactome Pathway	No. of Total Proteins in Pathway	No. of Hits in Pathway	*p*-Value	FDR Value	Hit Gene
Signaling by Interleukins	452	59	1.11 × 10^−16^	8.66 × 10^−15^	*APP*, *ATF2*, *MYC*, *AKT1*, *MIF*, *TP53*, *PIK3R1*, *HIF1A*, *STAT5A*, *STAT5B*, *JUN*, *GRB2*, *CDKN1A*, *IKBKB*, *JAK2*, *FOS*, *CTF1*, *PTGS2*, *RELA*, *STAT3*, *VEGFA*, *IRS1*, *IRS2*, *TNF*, *SQSTM1*, *SHC1*, *FOXO3*, *FOXO1*, *SOCS2*, *UBB*, *HSPA9*, *HSPA8*, *TGFB1*, *IL2*, *NFKB1*, *NFKB2*, *NFKBIA*, *IL6*, *IL7*, *BCL2*, *IL7R*, *HMGB1*, *PIK3CB*, *LMNB1*, *HSP90AA1*, *YWHAZ*, *CREB1*, *PIK3CA*, *IL2RG*, *CDC42*, *MAPK9*, *MAPK8*, *PTK2B*, *MAPK3*, *PTPN11*, *MAPK14*, *S100B*, *SOD2*, *SOD1*
DNA Double-Strand Break Repair	149	30	1.11 × 10^−16^	8.66 × 10^−15^	*CHEK2*, *TP53*, *PRKDC*, *TP53BP1*, *XRCC6*, *XRCC5*, *ATM*, *ATR*, *FEN1*, *BRCA1*, *BRCA2*, *PARP1*, *SIRT6*, *PCNA*, *WRN*, *RPA1*, *NBN*, *BLM*, *UBB*, *ABL1*, *UBE2I*, *SUMO1*, *H2AFX*, *CCNA2*, *MAPK8*, *POLD1*, *RAD52*, *RAD51*, *ERCC4*, *ERCC1*
PIP3 activates AKT signaling	286	46	1.11 × 10^−16^	8.66 × 10^−15^	*ATF2*, *AKT1*, *TP53*, *PDGFB*, *PIK3R1*, *EGR1*, *JUN*, *INSR*, *GRB2*, *CDKN1A*, *BMI1*, *PPARG*, *EGFR*, *RICTOR*, *IRS1*, *IRS2*, *PDGFRB*, *PDGFRA*, *FOXO4*, *FOXO3*, *FOXO1*, *UBB*, *FGF23*, *KL*, *ESR1*, *MDM2*, *FGFR1*, *GSK3B*, *GSK3A*, *PTEN*, *PIK3CB*, *PDPK1*, *NRG1*, *CREB1*, *PIK3CA*, *HDAC2*, *HDAC3*, *HDAC1*, *INS*, *ERBB2*, *MAPK3*, *EGF*, *PTPN11*, *MTOR*, *PML*, *STUB1*
Intracellular signaling by second messengers	326	49	1.11 × 10^−16^	8.66 × 10^−15^	*ATF2*, *AKT1*, *PRKCD*, *PRKCA*, *TP53*, *PDGFB*, *PIK3R1*, *EGR1*, *JUN*, *INSR*, *GRB2*, *CDKN1A*, *BMI1*, *PPARG*, *EGFR*, *ADCY5*, *RICTOR*, *IRS1*, *IRS2*, *PDGFRB*, *PDGFRA*, *FOXO4*, *FOXO3*, *FOXO1*, *UBB*, *FGF23*, *KL*, *ESR1*, *MDM2*, *FGFR1*, *GSK3B*, *GSK3A*, *PTEN*, *PIK3CB*, *PDPK1*, *NRG1*, *CREB1*, *PIK3CA*, *HDAC2*, *HDAC3*, *HDAC1*, *INS*, *ERBB2*, *MAPK3*, *EGF*, *PTPN11*, *MTOR*, *PML*, *STUB1*
Signaling by Receptor Tyrosine Kinases	493	60	1.11 × 10^−16^	8.66 × 10^−15^	*ATF2*, *AKT1*, *PRKCD*, *PRKCA*, *PDGFB*, *PIK3R1*, *APOE*, *STAT5A*, *EGR1*, *STAT5B*, *JUND*, *INSR*, *IGF2*, *IGF1*, *PTK2*, *GRB2*, *JAK2*, *HRAS*, *FOS*, *NCOR1*, *EGFR*, *RICTOR*, *STAT3*, *VEGFA*, *CTNNB1*, *IRS1*, *IRS2*, *IGF1R*, *EP300*, *PDGFRB*, *PDGFRA*, *SHC1*, *PSEN1*, *UBB*, *FGF23*, *KL*, *BDNF*, *ESR1*, *FGFR1*, *FLT1*, *PIK3CB*, *HSP90AA1*, *PDPK1*, *NRG1*, *NGF*, *CREB1*, *PIK3CA*, *INS*, *CDC42*, *ERBB2*, *PTK2B*, *MAPK3*, *PTPN1*, *EGF*, *PTPN11*, *MAPK14*, *S100B*, *MTOR*, *BAX*, *STUB1*
FOXO-mediated transcription	66	22	1.11 × 10^−16^	8.66 × 10^−15^	*AKT1*, *DDIT3*, *TXN*, *PCK1*, *PPARGC1A*, *CREBBP*, *CDKN1A*, *SIRT1*, *SIRT3*, *NR3C1*, *STK11*, *EP300*, *FOXO4*, *FOXO3*, *FOXO1*, *SIN3A*, *YWHAZ*, *CAT*, *HDAC2*, *HDAC1*, *INS*, *SOD2*
RNA Polymerase II Transcription	1363	110	1.11 × 10^−16^	8.66 × 10^−15^	*RB1*, *ATF2*, *MT-CO1*, *SERPINE1*, *CHEK2*, *MYC*, *AKT1*, *TP63*, *MED1*, *AR*, *DDIT3*, *TP53*, *TXN*, *PRDX1*, *APOE*, *PCK1*, *PPARGC1A*, *CREBBP*, *JUN*, *ATM*, *ATR*, *TP73*, *CDKN1A*, *BRCA1*, *BMI1*, *PARP1*, *FOS*, *SIRT1*, *SIRT3*, *NCOR2*, *NCOR1*, *PPARG*, *PPARA*, *PCNA*, *HTT*, *EGFR*, *RELA*, *WRN*, *RICTOR*, *CDKN2B*, *CDKN2A*, *GSR*, *RPA1*, *GTF2H2*, *MLH1*, *VEGFA*, *CTNNB1*, *FAS*, *NR3C1*, *CTGF*, *STK11*, *EP300*, *NBN*, *IGFBP3*, *BLM*, *MAX*, *FOXO4*, *FOXO3*, *FOXO1*, *UBB*, *ABL1*, *TFAP2A*, *TGFB1*, *UBE2I*, *BDNF*, *ESR1*, *IL2*, *NFKB1*, *CDK7*, *IL6*, *SP1*, *CDK1*, *MDM2*, *PIN1*, *TCF3*, *GSK3B*, *PTEN*, *SUMO1*, *SIN3A*, *TBP*, *PDPK1*, *H2AFX*, *YWHAZ*, *CCNA2*, *CREB1*, *TFDP1*, *CAT*, *CEBPB*, *HDAC2*, *HDAC3*, *HDAC1*, *PPM1D*, *HSPD1*, *INS*, *ERBB2*, *E2F1*, *MAPK3*, *PTPN1*, *PTPN11*, *MAPK14*, *SOD2*, *MTOR*, *PML*, *RAD51*, *ERCC3*, *SST*, *ERCC2*, *BAX*, *STUB1*, *TAF1*
Generic Transcription Pathway	1234	110	1.11 × 10^−16^	8.66 × 10^−15^	*RB1*, *ATF2*, *MT-CO1*, *SERPINE1*, *CHEK2*, *MYC*, *AKT1*, *TP63*, *MED1*, *AR*, *DDIT3*, *TP53*, *TXN*, *PRDX1*, *APOE*, *PCK1*, *PPARGC1A*, *CREBBP*, *JUN*, *ATM*, *ATR*, *TP73*, *CDKN1A*, *BRCA1*, *BMI1*, *PARP1*, *FOS*, *SIRT1*, *SIRT3*, *NCOR2*, *NCOR1*, *PPARG*, *PPARA*, *PCNA*, *HTT*, *EGFR*, *RELA*, *WRN*, *RICTOR*, *CDKN2B*, *CDKN2A*, *GSR*, *RPA1*, *GTF2H2*, *MLH1*, *VEGFA*, *CTNNB1*, *FAS*, *NR3C1*, *CTGF*, *STK11*, *EP300*, *NBN*, *IGFBP3*, *BLM*, *MAX*, *FOXO4*, *FOXO3*, *FOXO1*, *UBB*, *ABL1*, *TFAP2A*, *TGFB1*, *UBE2I*, *BDNF*, *ESR1*, *IL2*, *NFKB1*, *CDK7*, *IL6*, *SP1*, *CDK1*, *MDM2*, *PIN1*, *TCF3*, *GSK3B*, *PTEN*, *SUMO1*, *SIN3A*, *TBP*, *PDPK1*, *H2AFX*, *YWHAZ*, *CCNA2*, *CREB1*, *TFDP1*, *CAT*, *CEBPB*, *HDAC2*, *HDAC3*, *HDAC1*, *PPM1D*, *HSPD1*, *INS*, *ERBB2*, *E2F1*, *MAPK3*, *PTPN1*, *PTPN11*, *MAPK14*, *SOD2*, *MTOR*, *PML*, *RAD51*, *ERCC3*, *SST*, *ERCC2*, *BAX*, *STUB1*, *TAF1*
Cellular responses to stress	554	61	1.11 × 10^−16^	8.66 × 10^−15^	*RB1*, *ATF2*, *AR*, *DDIT3*, *TP53*, *TXN*, *HIF1A*, *PRDX1*, *CREBBP*, *JUN*, *ATM*, *ATR*, *CDKN1A*, *BMI1*, *FOS*, *TERF1*, *SIRT1*, *TERF2*, *GSTP1*, *RELA*, *HSF1*, *RAE1*, *CDKN2B*, *CDKN2A*, *IFNB1*, *GSR*, *STAT3*, *RPA1*, *VEGFA*, *HSPA1B*, *HSPA1A*, *NR3C1*, *EP300*, *NBN*, *MAP3K5*, *EEF1A1*, *VCP*, *UBB*, *HSPA9*, *HSPA8*, *NFKB1*, *IL6*, *SP1*, *MDM2*, *GSK3B*, *LMNB1*, *HSP90AA1*, *GPX1*, *H2AFX*, *CCNA2*, *TFDP1*, *CAT*, *CEBPB*, *MAPK9*, *MAPK8*, *E2F1*, *MAPK3*, *MAPK14*, *SOD2*, *MTOR*, *SOD1*
SUMO E3 ligases SUMOylate target proteins	169	36	1.11 × 10^−16^	8.66 × 10^−15^	*AR*, *TP53*, *TP53BP1*, *PPARGC1A*, *CREBBP*, *TOP2A*, *TOP2B*, *BRCA1*, *BMI1*, *PARP1*, *NCOR2*, *PPARG*, *PPARA*, *PCNA*, *RELA*, *WRN*, *RAE1*, *CDKN2A*, *RPA1*, *NR3C1*, *EP300*, *HIC1*, *BLM*, *TFAP2A*, *UBE2I*, *ESR1*, *NFKB2*, *NFKBIA*, *MDM2*, *SUMO1*, *SIN3A*, *TOP1*, *HDAC2*, *HDAC1*, *PML*, *RAD52*

**Table 3:** Reactome pathway enrichment analysis results—longevity genes.

Reactome Pathway	No. of Total Proteins in Pathway	No. of Hits in Pathway	*p*-Value	FDR	HitGenes
mTOR signalling	41	19	1.11 × 10^−16^	5.15 × 10^−14^	*AKT1*, *RRAGA*, *RRAGC*, *RRAGB*, *RRAGD*, *RPTOR*, *LAMTOR2*, *LAMTOR3*, *RPS6*, *TSC2*, *TSC1*, *EIF4EBP1*, *RHEB*, *MLST8*, *AKT1S1*, *EIF4E*, *EIF4B*, *MTOR*, *RPS6KB1*
mTORC1-mediated signalling	24	16	1.11 × 10^−16^	5.15 × 10^−14^	*RRAGA*, *RRAGC*, *RRAGB*, *RRAGD*, *RPTOR*, *LAMTOR2*, *LAMTOR3*, *RPS6*, *EIF4EBP1*, *RHEB*, *MLST8*, *AKT1S1*, *EIF4E*, *EIF4B*, *MTOR*, *RPS6KB1*
Intracellular signaling by second messengers	326	34	1.74 × 10^−14^	5.39 × 10^−12^	*AKT1*, *PRKCA*, *RRAGA*, *RRAGC*, *RRAGB*, *RRAGD*, *TP53*, *MAPKAP1*, *TGFA*, *INSR*, *CDKN1A*, *RPTOR*, *PPARG*, *EGFR*, *RICTOR*, *CAMK4*, *LAMTOR2*, *LAMTOR3*, *IRS2*, *TSC2*, *FOXO4*, *FOXO3*, *FOXO1*, *KL*, *ESR1*, *NBEA*, *RHEB*, *FGFR1*, *PRR5*, *MLST8*, *PIK3CA*, *AKT1S1*, *INS*, *MTOR*
PIP3 activates AKT signaling	286	31	1.03 × 10^−13^	2.39 × 10^−11^	*AKT1*, *RRAGA*, *RRAGC*, *RRAGB*, *RRAGD*, *TP53*, *MAPKAP1*, *TGFA*, *INSR*, *CDKN1A*, *RPTOR*, *PPARG*, *EGFR*, *RICTOR*, *LAMTOR2*, *LAMTOR3*, *IRS2*, *TSC2*, *FOXO4*, *FOXO3*, *FOXO1*, *KL*, *ESR1*, *RHEB*, *FGFR1*, *PRR5*, *MLST8*, *PIK3CA*, *AKT1S1*, *INS*, *MTOR*
Generic Transcription Pathway	1234	66	3.56 × 10^−13^	6.59 × 10^−11^	*SERPINE1*, *AKT1*, *RUNX3*, *RRAGA*, *RRAGC*, *RRAGB*, *RRAGD*, *TP53*, *MAPKAP1*, *TGFA*, *GATA4*, *APOE*, *ATRIP*, *PPARGC1A*, *ATM*, *CDKN1A*, *MSTN*, *RPTOR*, *SREBF1*, *SIRT1*, *SIRT3*, *RAD51D*, *RARB*, *PPARG*, *SGK1*, *EGFR*, *WRN*, *RICTOR*, *WWOX*, *CDKN2B*, *GSR*, *MLH1*, *VEGFA*, *CAMK4*, *FAS*, *LAMTOR2*, *LAMTOR3*, *NR3C1*, *YY1*, *KCTD1*, *TSC2*, *TSC1*, *TBL1XR1*, *CSF1R*, *FOXO4*, *FOXO3*, *FOXO1*, *PLXNA4*, *TGFB1*, *ESRRG*, *ESR1*, *NFKB1*, *IL6*, *CDK6*, *RHEB*, *PRR5*, *EXO1*, *MLST8*, *YWHAG*, *H2AFX*, *IFNG*, *INS*, *TXNRD1*, *SOD2*, *MTOR*, *ERCC2*
Energy dependent regulation of mTOR by LKB1-AMPK	29	12	2.20 × 10^−12^	3.39 × 10^−10^	*RRAGA*, *RRAGC*, *RRAGB*, *RRAGD*, *RPTOR*, *LAMTOR2*, *LAMTOR3*, *TSC2*, *TSC1*, *RHEB*, *MLST8*, *MTOR*
RNA Polymerase II Transcription	1363	67	1.04 × 10^−11^	1.29 × 10^−9^	*SERPINE1*, *AKT1*, *RUNX3*, *RRAGA*, *RRAGC*, *RRAGB*, *RRAGD*, *TP53*, *MAPKAP1*, *TGFA*, *GATA4*, *APOE*, *ATRIP*, *PPARGC1A*, *ATM*, *CDKN1A*, *MSTN*, *RPTOR*, *SREBF1*, *SIRT1*, *SIRT3*, *RAD51D*, *RARB*, *PPARG*, *SGK1*, *EGFR*, *WRN*, *RICTOR*, *WWOX*, *CDKN2B*, *GSR*, *MLH1*, *VEGFA*, *CAMK4*, *FAS*, *LAMTOR2*, *LAMTOR3*, *NR3C1*, *YY1*, *KCTD1*, *TSC2*, *TSC1*, *TBL1XR1*, *CSF1R*, *FOXO4*, *FOXO3*, *FOXO1*, *PLXNA4*, *TGFB1*, *ESRRG*, *ESR1*, *NFKB1*, *IL6*, *CDK6*, *RHEB*, *PRR5*, *EXO1*, *MLST8*, *YWHAG*, *H2AFX*, *IFNG*, *POLDIP3*, *INS*, *TXNRD1*, *SOD2*, *MTOR*, *ERCC2*
TP53 Regulates Metabolic Genes	89	17	1.11 × 10^−11^	1.29 × 10^−9^	*AKT1*, *RRAGA*, *RRAGC*, *RRAGB*, *RRAGD*, *TP53*, *RPTOR*, *GSR*, *LAMTOR2*, *LAMTOR3*, *TSC2*, *TSC1*, *RHEB*, *MLST8*, *YWHAG*, *TXNRD1*, *MTOR*
FOXO-mediated transcription	66	15	1.73 × 10^−11^	1.78 × 10^−9^	*AKT1*, *PPARGC1A*, *CDKN1A*, *MSTN*, *SREBF1*, *SIRT1*, *SIRT3*, *NR3C1*, *FOXO4*, *FOXO3*, *FOXO1*, *PLXNA4*, *YWHAG*, *INS*, *SOD2*
Transcriptional Regulation by TP53	368	30	2.68 × 10^−10^	2.25 × 10^−8^	*AKT1*, *RRAGA*, *RRAGC*, *RRAGB*, *RRAGD*, *TP53*, *MAPKAP1*, *ATRIP*, *ATM*, *CDKN1A*, *RPTOR*, *RAD51D*, *SGK1*, *WRN*, *RICTOR*, *GSR*, *MLH1*, *FAS*, *LAMTOR2*, *LAMTOR3*, *TSC2*, *TSC1*, *RHEB*, *PRR5*, *EXO1*, *MLST8*, *YWHAG*, *TXNRD1*, *MTOR*, *ERCC2*

**Table 4:** Comparison of AR and positive AD genes.

GenAge (AR) Genes	AlzGene (AD) Genes	Overlap Genes	Overlapped in AR Genes (%)
307	356	41	13%

**Table 5:** Reactome pathway enrichment analysis results—AD–AR overlap genes.

Reactome Pathway	No. of Total Proteins in Pathway	No. of Hits in Pathway	*p*-Value	FDR Value	Hit Gene
Generic Transcription Pathway	1234	22	4.05 × 10^−11^	1.85 × 10^−8^	*GSK3B*, *MT-CO1*, *SERPINE1*, *APOE*, *PCK1*, *TP63*, *TGFB1*, *UBE2I*, *PARP1*, *BDNF*, *CDKN2A*, *SOD2*, *ESR1*, *AR*, *IL6*, *SST*, *FAS*, *PIN1*, *PPARG*, *PPARA*, *TP53*, *TP73*
RNA Polymerase II Transcription	1363	22	2.84 × 10^−10^	6.47 × 10^−8^	*GSK3B*, *MT-CO1*, *SERPINE1*, *APOE*, *PCK1*, *TP63*, *TGFB1*, *UBE2I*, *PARP1*, *BDNF*, *CDKN2A*, *SOD2*, *ESR1*, *AR*, *IL6*, *SST*, *FAS*, *PIN1*, *PPARG*, *PPARA*, *TP53*, *TP73*
SUMOylation of intracellular receptors	30	5	1.08 × 10^−7^	1.62 × 10^−5^	*UBE2I*, *ESR1*, *AR*, *PPARG*, *PPARA*
TP53 Regulates Transcription of Death Receptors and Ligands	12	4	1.42 × 10^−7^	1.62 × 10^−5^	*TP63*, *FAS*, *TP53*, *TP73*
SUMO E3 ligases SUMOylate target proteins	169	8	1.98 × 10^−7^	1.80 × 10^−5^	*UBE2I*, *PARP1*, *CDKN2A*, *ESR1*, *AR*, *PPARG*, *PPARA*, *TP53*
Signaling by ERBB4	58	5	2.69 × 10^−6^	1.75 × 10^−4^	*PIK3R1*, *PSEN1*, *APOE*, *S100B*, *ESR1*
Interleukin-4 and Interleukin-13 signaling	112	6	3.83 × 10^−6^	2.18 × 10^−4^	*PIK3R1*, *PTGS2*, *TNF*, *TGFB1*, *IL6*, *TP53*
Apoptosis	179	7	4.36 × 10^−6^	2.18 × 10^−4^	*LMNA*, *TP63*, *CDKN2A*, *FAS*, *MAPT*, *TP53*, *TP73*
Nuclear signaling by ERBB4	32	4	6.83 × 10^−6^	2.80 × 10^−4^	*PSEN1*, *APOE*, *S100B*, *ESR1*
Platelet degranulation	128	6	8.16 × 10^−6^	3.10 × 10^−4^	*APP*, *SERPINE1*, *CLU*, *A2M*, *TGFB1*, *IGF1*

**Table 6:** Comparison of Longevity and AD Genes.

Longevity Genes	Positive AlzGene (AD) Genes	Overlap Genes	Overlapped in Longevity Genes %
357	356	43	12%

**Table 7:** Reactome pathway enrichment analysis results—longevity and AD overlap genes.

Reactome Pathway	No. of Total Proteins in Pathway	No. of Hits in Pathway	*p*-Value	FDR Value	Hit Gene
Interleukin-4 and Interleukin-13 signaling	112	7	7.64 × 10^−8^	2.32 × 10^−5^	*TNF*, *HMOX1*, *IL10*, *TGFB1*, *IL18*, *IL6*, *TP53*
Plasma lipoprotein assembly	19	4	5.15 × 10^−7^	7.82 × 10^−5^	*APOE*, *APOA1*, *APOA4*, *APOC1*
Plasma lipoprotein assembly, remodeling, and clearance	71	5	3.69 × 10^−6^	2.30 × 10^−4^	*CETP*, *APOE*, *APOA1*, *APOA4*, *APOC1*
Plasma lipoprotein remodeling	32	4	4.02 × 10^−6^	2.30 × 10^−4^	*CETP*, *APOE*, *APOA1*, *APOA4*
Chylomicron remodeling	10	3	5.35 × 10^−6^	2.30 × 10^−4^	*APOE*, *APOA1*, *APOA4*
Chylomicron assembly	10	3	5.35 × 10^−6^	2.30 × 10^−4^	*APOE*, *APOA1*, *APOA4*
HDL remodeling	10	3	5.35 × 10^−6^	2.30 × 10^−4^	*CETP*, *APOE*, *APOA1*
Signaling by Interleukins	452	9	1.20 × 10^−5^	4.57 × 10^−4^	*AGER*, *TNF*, *HMOX1*, *IL10*, *TGFB1*, *IL18*, *SOD2*, *IL6*, *TP53*
Retinoid metabolism and transport	44	4	1.40 × 10^−5^	4.61 × 10^−4^	*TTR*, *APOE*, *APOA1*, *APOA4*
Interleukin-10 signaling	47	4	1.80 × 10^−5^	5.29 × 10^−4^	*TNF*, *IL10*, *IL18*, *IL6*

## Data Availability

All data are available within the manuscript.

## Supplementary Materials

The supporting information can be downloaded at: https://www.mdpi.com/article/10.3390/ijms24065178/s1.
